# The impact of intervention strategies that target arterial stiffness in end-stage renal disease: a systematic review protocol

**DOI:** 10.1186/s13643-016-0286-5

**Published:** 2016-07-19

**Authors:** Rosendo A. Rodriguez, Beverley Shea, Richard Hae, Kevin D. Burns

**Affiliations:** Department of Medicine, The Ottawa Hospital and University of Ottawa, 501 Smyth Road, Ottawa, Ontario K1H 8L6 Canada; The Ottawa Methods Centre, Ottawa Hospital Research Institute, Centre for Practice Changing Research Building, 501 Smyth Road, Ottawa, Ontario K1H 8L6 Canada; Knowledge Synthesis Group, Ottawa Hospital Research Institute, Centre for Practice Changing Research Building, 501 Smyth Road, Ottawa, Ontario K1H 8L6 Canada; Division of Nephrology, Kidney Research Centre, Ottawa Hospital Research Institute, University of Ottawa, 1967 Riverside Drive, Ottawa, Ontario K1H 7W9 Canada

**Keywords:** End-stage kidney disease, Arterial or vascular Stiffness, Risk factors, Cardiovascular diseases, Stroke

## Abstract

**Background/objectives:**

Vascular damage contributes to the high cardiovascular morbidity and mortality in end-stage renal disease (ESRD). Increased aortic stiffness measured by carotid-femoral pulse wave velocity (cf-PWV) is a strong and independent predictor of the cardiovascular risk in ESRD patients. Recently, there has been considerable interest in developing strategies to lessen the progression of arterial stiffness in ESRD patients using cf-PWV as a tool to monitor therapeutic responses, but their benefit on the long-term cardiovascular risk is not known. Appraisal of the effects of existing stiffness-based interventions on the cf-PWV would facilitate selecting optimal therapies to be tested in randomized clinical trials. The aim of this systematic review will be to evaluate the impact of arterial stiffness-based interventions on the cf-PWV in ESRD patients. Secondarily, for each intervention, we will determine the minimal duration needed to achieve a significant reduction of cf-PWV, the minimal cf-PWV reduction threshold or effect size, and adverse events.

**Methods/design:**

This review will be conducted using MEDLINE, EMBASE, and EBM Reviews. We will select clinical trials and observational studies (cohort, case-control, and before/after studies and case series) that evaluated pharmacologic or non-pharmacologic interventions in which the primary effect is to improve structural and/or dynamic components of arterial stiffness in adults with stage 5 chronic kidney disease. The primary outcome of interest will be cf-PWV. Study selection and data collection will be performed by two reviewers. Validated tools will be used to assess the methodological quality and risk of bias among different study designs. We will describe all included citations according to study characteristics, methodological quality, and outcomes. Suitability for meta-analysis will be determined by the degree of clinical and statistical heterogeneity between studies. If appropriate, we will calculate effect estimates by obtaining the relative risks with 95 % confidence intervals pooled according to study design using a random effects model.

**Discussion:**

This review will summarize evidence regarding effects of interventions targeting arterial stiffness in ESRD patients. Our results will inform clinicians and researchers on the type of existing arterial stiffness-based interventions for ESRD patients and their potential efficacy and safety, with a goal to guide future clinical trials aimed at reducing adverse cardiovascular events.

**Systematic review registration:**

PROSPERO CRD42016033463

**Electronic supplementary material:**

The online version of this article (doi:10.1186/s13643-016-0286-5) contains supplementary material, which is available to authorized users.

## Background

Chronic kidney disease (CKD) is a major health burden worldwide because of its high prevalence and associated risk of end-stage renal disease (ESRD), cardiovascular disease, and premature death. The age-standardized global prevalence of CKD (stages 1–5) among adults (≥20 years old) has been reported to be approximately 10.4 % for men (95 % CI, 9.3–11.9 %) and 11.8 % for women (95 % CI, 11.2–12.6 %) [1]. In 2013, 956,200 deaths worldwide were reported to be directly attributed to CKD, representing a 134.6 % increase from those reported in 1990 [2]. Additionally, the high prevalence of CKD has led to an increased prevalence of ESRD worldwide and elevated health care costs. It is estimated that in the period from 2000 to 2007, the total direct Medicare costs for ESRD in the USA alone increased from $ 12.2 billion to $ 20.8 billion [3]. In Canada, the number of people with ESRD in the last decade rose from 29,540 to 41,252, representing an increase of 40 % [4]. By the end of 2013, 23,926 Canadians with ESRD were receiving some form of dialysis, but only 15 % of those were on the waiting list for a renal transplant [4].

ESRD increases the risk of death by approximately 10 to 20 times compared to the general population [5]. Cardiovascular disease accounts for almost 50 % of all deaths in ESRD [5, 6]. Although occlusive thrombotic coronary artery disease is common during the early stages of CKD, in those who survive to reach ESRD requiring dialysis, sudden death due to the presence of arrhythmias, myocardial ischemia, and heart failure is not uncommon [7, 8]. This phenomenon is associated with accelerated atherosclerosis and vascular calcification that leads to a loss of arterial compliance and increased *arterial stiffness*, which in turn increases left ventricle overload and decreases sub-endocardial perfusion [9, 10].

Arterial stiffness, defined as a decreased ability of the conductive arteries to absorb the pulse pressure has been reported to be a strong independent predictor of overall cardiovascular mortality in the general population [11, 12]. Since the aorta is the principal capacitive element of the arterial tree, non-invasive measurements of stiffness in the aorta obtained by applanation tonometry using the carotid-femoral pulse wave velocity (cf-PWV) can more accurately reflect the physiologic effects of increased arterial stiffness on the heart [13, 14]. Indeed, elevated cf-PWV in ESRD patients who are treated with peritoneal dialysis, hemodialysis, or renal transplantation is an excellent predictor of fatal cardiovascular events and all-cause mortality [15–19]. An increase of 1 m/s in cf-PWV in ESRD patients increases both cardiovascular and overall mortality by 34 % by crude estimates and by 14 % after adjusted analyses [20]. After adjustment for potential confounders, ESRD patients who have cf-PWV greater than or equal to 12.0 m/s are five to six times more likely to die compared to those patients with values less than 9.4 m/s [21].

Because of the excessive burden of cardiovascular disease in ESRD patients, there has been considerable interest in developing strategies to lessen or delay the progression of arterial stiffness in this high-risk group [9, 22], using cf-PWV as a tool to monitor therapeutic responses [11, 12]. The benefit of these interventions, however, on the long-term cardiovascular risk of ESRD patients is not known. A systematic appraisal of the effects of existing stiffness-based interventions on the cf-PWV would facilitate the selection of optimal therapies to be tested in large randomized clinical trials. Accordingly, we will conduct a systematic review of studies that have explored the potential benefit of interventions on arterial stiffness in humans with ESRD. Our knowledge synthesis will examine the current clinical evidence on the impact of interventions in ESRD patients directed at reducing arterial stiffness, based on measures of cf-PWV.

## Objectives

The primary objective of this review is to evaluate the impact of stiffness-based therapeutic interventions on the reduction of cf-PWV in ESRD patients. Secondary objectives of our review will include the following: (1) to determine the minimal duration of each intervention needed to achieve a reduction of cf-PWV; (2) to define a minimal cf-PWV reduction threshold or effect size for each therapeutic option; and (3) to describe any reported serious or non-serious adverse events associated with the use of each intervention.

## Methods and design

This review will be conducted in accordance with The Cochrane Collaboration principles for Systematic Reviews [23] and reported following the Preferred Reporting Items for Systematic Review and Meta-Analysis (PRISMA) guidelines [24] and the PRISMA for abstracts checklist [25]. The content of this protocol follows the PRISMA Protocols (PRISMA-P) recommendations [26]. The checklist of the PRISMA-P recommendations is included as an additional file (see Additional file 1). The review protocol has been registered in PROSPERO International Prospective Register of Systematic Reviews (CRD42016033463) [27].

## Eligibility criteria

### Study population

We will include adults (older than 18 years old) with severely reduced kidney function (ESRD/CKD stage 5; with an estimated glomerular filtration rate of less than 15 ml/min/1.73 m^2^) of any duration receiving or not receiving renal replacement therapy. Kidney transplant recipients who are not on dialysis therapy will be included if they had pre- and post-transplant assessments of their cf-PWV.

### Types of intervention

In ESRD patients, arterial stiffening develops from a complex interaction between stable and dynamic changes involving structural elements of the vessel wall as a result of different factors such as increased wall stress from hypertension, augmented extra-cellular matrix collagen content, diffuse calcification, and vascular smooth-muscle cell proliferation [28]. In this systematic review, we will incorporate any intervention in which the primary effect is to improve the structural (qualitative and/or quantitative) and/or dynamic components of arterial stiffness [22, 28]. This will include pharmacologic interventions aimed at controlling blood pressure, vascular inflammation, and arterial resistance (i.e., endothelin-A receptor antagonists) or the plasma antioxidant status (i.e., vitamin E analogues), to limit the progression of vascular calcification (i.e., calcium-sensing receptor-inhibitors, agents that restore calcium/phosphorous imbalance) or atherosclerosis (i.e., 3-hydroxy-3-methylglutaryl-coenzyme A reductase inhibitors) or to improve extra-cellular fluid volume fluctuations (i.e., frequency/duration of dialysis, changes in dialysate components, bio-impedance-guided fluid volume control), renal replacement therapy (i.e., peritoneal dialysis, hemodialysis, kidney transplantation), advanced glycation end product inhibitors (i.e., aminoguanidine, alagebrium chloride), and other non-pharmacologic therapies with potential protective cardiovascular effects (i.e., physical fitness, nutrition, exercise) [22, 28].

### Comparators

There will be no restrictions on the type of comparator. Placebo, supportive, or standard care and other therapeutic interventions will be accepted.

### Outcomes

The primary outcome of interest will be cf-PWV defined by arterial pulse wave measurements on the carotid and femoral arteries in studies that have used previously validated instrumentation. Secondary outcomes will include the following estimates associated with each intervention: (a) minimal duration needed to achieve a significant reduction of cf-PWV; (b) minimal cf-PWV reduction threshold or effect size; and (c) any reported serious or non-serious adverse events. We will consider a serious adverse event as any untoward medical occurrence associated with the intervention that at any dose may result in (a) death; (b) a life-threatening condition; (c) in-patient hospitalization or prolongation of existing hospitalization; (d) persistent or significant disability/incapacity; or (e) a subsequent intervention to prevent permanent functional impairment or damage [29].

### Study design

Prospective clinical trials and observational studies (cohort, case-control, and before and after studies and case series) will be included provided that 10 or more ESRD participants have received the intervention and its effects were assessed by cf-PWV.

### Languages

We will include articles reported in the English, French, Italian, and Spanish languages. A list of possibly relevant titles in any other language will be provided as an appendix.

## Search strategy

Our search strategy will be conducted using MEDLINE, EMBASE, Cochrane Central Databases, CADTH’s “Grey Matters Light”, OVID, EBM Reviews, Health Technology Assessment (HTA) database, and the gray literature of studies published between January 1965 and March 2016 (see Additional file 2). EMBASE also includes the abstract publications from major international conferences including International, American, Canadian, Australian, and New Zealand Societies of Nephrology as well as the International, European, and Canadian Societies of Hypertension. A comprehensive search strategy will be constructed and implemented by a health information specialist with systematic review experience, in collaboration with the research team. MeSH terms will be used to capture each of the principal elements of the research question. Identified citations will be exported to a citation manager for study selection. Manual review of the reference lists of all included studies and previous systematic reviews according to our pre-defined screening criteria will be conducted. The latter will be used as an additional source of primary studies. A final gray literature search will be conducted using “Google Scholar” as well as reviews of *clinicaltrials.gov* for any current study. No language restriction will be utilized in any of the initial searches, but our final analysis will be restricted to citations published in English, French, Spanish, or Italian. Duplicate citations will be removed, and search strategies will be kept up to the time of the end of the review.

## Study screening and exclusions

Clinical trials and observational studies (cohort, case-control, and before and after studies and case series) that include stiffness-based interventions in ESRD patients will be considered. Studies will also be considered if there has been previous exposure of ESRD patients to an intervention and if cf-PWV is one of the study outcomes. An iterative process for study selection will be followed using the inclusion and exclusion criteria set out in Table 1. Specifically, the screening phase will include all prospective and retrospective studies that report the effects of an intervention on arterial stiffness (measured by cf-PWV) in adult ESRD patients (age ≥18 years). We will exclude studies that exclusively report on adult CKD stages 1 to 4 or pediatric populations (age <18 years), those that only include kidney transplant patients without any pre-transplant assessment of the cf-PWV, non-human studies, narrative reviews, in vitro or mathematical modeling reports, and any duplicate or sub-study of previously published trials. Studies that exclusively report on different markers of arterial stiffness such as brachial-ankle PWV, ankle-brachial index, augmentation index, carotid-brachial PWV, and femoral-tibial PWV will not be included in this review. All citations will first be screened by title and abstract. This process will be performed in duplicate by two reviewers, and a third one for consensus in cases of discrepancies. To facilitate the review at a text level, an intermediate screening phase will be performed and the methods section of each paper retrieved from the “title and abstract” screening phase will be reviewed to confirm that cf-PWV was the technique used to measure aortic stiffness, that an appropriate instrumentation was used, that ESRD patients were included, and that the characteristics of an intervention were well documented. The intermediate screening will be performed by one of the authors (RAR) with a second reviewer independently checking for discrepancies in only 20 % of the files. All citations not excluded by the initial and intermediate screens will have full articles retrieved for a subsequent review, in duplicate by two independent reviewers, and the final selection criteria of Table 1 applied. Any differences in classification between the two independent reviewers will be reviewed and consensus decision made. We will record the reasons for excluding trials. A third independent author will be asked to provide an independent opinion in all instances in which consensus is not reached. Then, a judicious decision will be made based on the option proposed by the independent reviewer. In case of uncertainties on missing information about treatments, methodology, and/or outcomes of included studies, we will consider contacting study authors. Neither of the review authors will be blind to the journal titles or to the study authors or institutions.

**Table 1:** Study selection criteria

Descriptor	Inclusion criteria	Exclusion criteria
Patient population	Adults (≥18 years old) with end-stage renal disease (ESRD) defined as stage 5 chronic kidney disease (e-GFR <15 ml/min/1.73 m^2^) of any duration and receiving or not any renal replacement therapy. Kidney transplant recipients who are not on dialysis therapy will be included if they had pre- and post-transplant assessments of their cf-PWV.	Chronic kidney disease stages 1 to 4 Population-based studies that do not include ESRD patients Non-human studies Pediatric populations (<18 years of age) Kidney transplant patients without any assessment of carotid-femoral pulse wave velocity before transplantation
Intervention	Any pharmacologic or non-pharmacologic intervention whose primary effect is to improve the structural (qualitative and quantitative) and/or dynamic components of arterial stiffness. This will include interventions aimed to control blood pressure or vascular inflammation, to limit the progression of vascular calcification or atherosclerosis, and to improve extra-cellular volume fluctuations, renal replacement therapy, and advanced glycation end products.	
Comparator	A different intervention, placebo, control group, or patients exposed to standard care ESRD management	
Outcome	Primary outcome: reduction on the carotid-femoral pulse wave velocity defined by pulse wave measurements on the carotid and femoral arteries using previously validated instrumentation and proper methodological technique. Secondary outcomes will include the following estimates associated with each intervention: (a) minimal duration needed to achieve a significant reduction of cf-PWV; (b) minimal cf-PWV reduction threshold or effect size; and (c) any reported serious or non-serious adverse events.	Studies that exclusively report on different markers of arterial stiffness such as brachial-ankle PWV, ankle-brachial index, augmentation index, carotid-brachial PWV and femoral-tibial PWV
Study design	Clinical trials and observational studies (cohort, case-control, and before and after studies and case series) (both prospective and retrospective) provided that 10 or more ESRD participants have received the intervention and its effects were assessed by cf-PWV)	Reviews, in vitro studies, mathematical models, duplicates or “sub-cohorts” of previously published cohorts
Language	English, French, Spanish, Italian	

*ESRD* end-stage renal disease, *cf-PWV* carotid-femoral pulse wave velocity, *e-GFR* estimated glomerular filtration rate, *PWV* pulse wave velocity

## Data extraction

A data extraction form will be prepared a priori through consensus from all investigators and piloted prior to duplicate extraction by two independent reviewers. Data extraction will include (a) *study characteristics, design, and methods*: title, authors, journal/source/year and language of publication, country, type of study design, study period, publication status, total number of patients, case ascertainment and/or inclusion/exclusion criteria, single or multicenter study, randomization, allocation concealment, and blinding methods (where applicable); (b) *sample characteristics*: age, sex, type of renal replacement therapy, dialysis vintage and co-morbidities, duration of follow-up, and type of arterial stiffness instrumentation used; (c) *interventions and co-interventions*: type of pharmacologic or non-pharmacologic therapy utilized to limit the progression of arterial stiffness, its mechanism of action on the components (structural and/or dynamic) of arterial stiffness [22, 28], generic and trade names of the experimental intervention, dose, frequency and duration of treatment, type of comparator and its dose, and duration of follow-up; and (d) *outcomes*: reduction on cf-PWV as primary outcome and the criteria used in the definition of the effect size and its units of measurement. We will also record the duration for each stiffness-based therapy (when available), any reported threshold of cf-PWV to differentiate between high and low PWV, and clinical complications including mortality and other adverse events.

### Analysis plan

A description of all included studies, including demographic, clinical, and methodological quality will be first reported with the aid of tables and text. This narrative synthesis will explain the relationships and findings within and between the included studies in line with the guidance from the Centre for Reviews and Dissemination [24]. Suitability for meta-analysis will be determined by the degree of heterogeneity (clinical and statistical) observed between the studies. Statistical heterogeneity will be described using the *I*^2^ statistic. If meta-analysis is feasible, we will calculate the effect measures by obtaining the relative risks with their 95 % confidence intervals and pooled according to study design (randomized trials and observational studies, respectively) using a random effects model.

### Primary outcome

We anticipate that the primary outcome (reduction in cf-PWV) may be reported differently according to the study design. Some studies may report absolute or relative values that express the magnitude of reduction in cf-PWV by the intervention while others may express the results based on the proportion of patients above or below a certain critical cf-PWV threshold. Results will be expressed as continuous or dichotomous measures depending on the reported outcome measure. Summaries of continuous data will be presented as means and mean differences with 95 % confidence intervals. Given that a certain degree of heterogeneity is expected, a random effects model will be used if we proceed to conduct a meta-analysis. Statistical heterogeneity will be reported using the *I*^2^ test with 95 % confidence interval.

### Secondary outcomes

Secondary outcomes will be a combination of dichotomous, ordinal, and continuous measures. All continuous outcome variables will be described with means or medians and associated standard deviations or interquartile ranges as appropriate. Summaries of continuous data will be presented as means and mean differences with 95 % confidence intervals.

## Risk of bias

Risk of bias will be assessed using the “SIGN50” tool [30] for observational studies (cohort and case-control studies) and the Cochrane Collaboration tool for assessing the risk of bias in randomized clinical trials [31]. The assessment of the risk of bias will be performed in duplicate by two independent evaluators. Any discrepancy not resolved by consensus will be reviewed by a third senior author. Risk of bias assessment of all included studies will be summarized and presented in table format. If possible, we will perform meta-analysis with a planned sensitivity analysis using only those studies with low risk of bias and high methodological quality. Low risk of bias will be defined as those studies where the majority of criteria for methodological quality are met with little or no risk of bias (“++”) using the SIGN50 tool or those deemed low risk across all domains of the Cochrane’s collaboration tool for assessing risk of bias. For observational studies, criteria will include quality of data reporting, high internal and external validity, low risk of confounding, and sufficient study power. If there are sufficient number of trials (i.e., ≥10 studies), we will construct a funnel plot to assess for possible publication bias.

### Sub-group analyses

Pre-planned sub-group analysis to examine clinical heterogeneity will include, if possible, dialysis vintage (≥2 vs <2 years), diabetes mellitus (diabetic vs non-diabetics), age subgroups (≥50 vs <50 years), and type of renal replacement therapy (pre-dialysis, peritoneal dialysis, conventional hemodialysis, frequent hemodialysis, and renal transplantation).

### Sensitivity analyses

To improve the robustness of our findings, our pre-planned sensitivity analyses will include the following: (a) studies with low versus high/unclear risk of bias; (b) studies conducted after 2005 (when arterial stiffness was recognized by a consensus of international experts as a target for therapeutic options aimed to improve cardiovascular outcomes) [32, 33]; and (c) studies with follow-up ≥12 months (as a sustained effect of the intervention is expected).

### Confidence in cumulative estimate

If we find sufficient information in the literature for the different types of stiffness-based interventions, we will attempt to apply the GRADE methodology to rate the quality of the body of evidence and recommendations as high, moderate, low, or very low quality [34].

### Amendments

In the event of protocol amendments, we will provide the date of each amendment in a tabular form with the description of the changes and rationale.

## Discussion

Increased arterial stiffness exerts a number of adverse effects on cardiovascular function and is associated with heightened mortality in patients with ESRD [10, 14, 16, 18, 20, 21, 35]. Thus, the prevention and treatment of elevated arterial stiffness is important in this high-risk population. Evidence indicates that certain strategies whose mechanism targets the dynamic and/or structural components of vascular stiffness appear to be clinically efficacious interventions for preventing the progression of arterial stiffness [9, 10, 22, 28, 33, 35]. These interventions seem to have variable effectiveness in ESRD patients and are often not clearly defined [22, 28]. Whether combined stiffness-based therapies would provide better survival benefit compared with single interventions in ESRD patients, it will require testing in large randomized controlled trials. Thus, a systematic appraisal of existing stiffness-based interventions would facilitate selection of optimal therapies to be evaluated in those clinical trials.

This review proposes to systematically identify, assemble, and summarize the existing evidence regarding the effects of pharmacologic and non-pharmacological interventions targeting aortic stiffness in ESRD patients. We will also summarize evidence on the duration of interventions and the minimal cf-PWV reduction threshold, effect size, and adverse events associated with each intervention. These results will inform decisions affecting the design of clinical trials and will serve as a summation of the evidence to guide clinical decisions at the bedside.

Since we expect that the majority of available evidence will have been generated by observational and cohort studies rather than randomized controlled trials, we have planned for a large descriptive component of this review. To minimize the impact of potential risk of bias in these studies, we have incorporated validated tools that will assess the methodological quality and risk of bias among cohort and case-control studies, in addition to measures of clinical and statistical heterogeneity. Two recent meta-analyses have provided strong evidence that aortic PWV improves risk prediction for cardiovascular disease and mortality in the general population, beyond conventional risk factors [11, 12]. Most studies included in the analysis were observational and performed in hypertensive patients or the general population, except for three studies in ESRD patients. These analyses indicated that the pooled age and sex adjusted hazard ratio (HR) for cardiovascular events per 1-SD change in the cf-PWV was 1.35 (95 % CI, 1.22 to 1.50) for coronary artery disease, 1.54 (95 % CI, 1.34–1.78) for stroke, and 1.45 (95 % CI, 1.30–1.61) for cardiovascular disease. A sub-group analysis confirmed that an elevated cf-PWV in the presence of chronic kidney disease (stages 3 to 5) increased the risk for cardiovascular events by 48 % (HR, 1.48; 95 % CI, 1.24–1.76). Thus, these results support the notion that cf-PWV may be a suitable target for novel risk reduction strategies and that attenuation on the cf-PWV could be associated with improved survival.

Acceptance of cf-PWV as a useful biomarker for guiding risk stratification and/or treatment in ESRD depends on providing convincing evidence through randomized controlled trials. Our proposed knowledge synthesis will not only inform clinicians and researchers as end-users on the types and effects of existing interventions that target regression of arterial stiffness in ESRD but will also provide information on the efficacy and safety of these interventions to guideline producers and health policy makers. This review will also highlight weaknesses in study design and thereby inform future studies to reduce arterial stiffness with a goal to prevent adverse cardiovascular events in ESRD patients.

## Abbreviations

CKD, chronic kidney disease; cf-PWV, carotid-femoral pulse wave velocity; ESRD, end-stage renal disease; PWV, pulse wave velocity

## Additional files

Additional file 1: PRISMA-P checklist. Adapted checklist for use with protocol submissions to *Systematic Reviews.* (DOCX 37.3 kb) Additional file 2: Sample search strategy. Draft of search strategy from electronic databases including EMBASE, OVID MEDLINER, and other non-indexed citations with planned limits. (DOCX 23.9 kb)
