# Supplementary material for: The impact of intervention strategies that target arterial stiffness in end-stage renal disease: a systematic review protocol
Source: Syst Rev. 2016 Jul 19;5:118. doi: 10.1186/s13643-016-0286-5 (PMC4950258; doi:10.1186/s13643-016-0286-5)
Supplement: Additional file 2: — Sample search strategy. Draft of search strategy from electronic databases including EMBASE, OVID MEDLINER, and other non-indexed citations with planned limits. (DOCX 23.9 kb) [file 13643_2016_286_MOESM2_ESM.docx]

**Appendix 1**

**Sample Search Strategy**

Database: Embase Classic+Embase <1965 to 2016 March 31>, Ovid MEDLINE(R) In-Process & Other Non-Indexed Citations and Ovid MEDLINE(R) <1946 to Present> Search Strategy:

--------------------------------------------------------------------------------

1 exp Renal Insufficiency, Chronic/ (138762)

2 exp Kidney Diseases/ and Chronic Disease/ (22043)

3 ((kidney* or renal) adj1 (disease* or failure* or impair* or insufficien*)).tw. (397987)

4 (ESRD or ESKD or ESRF or CKD).tw. (55275)

5 exp Renal Dialysis/ (231814)

6 (dialys#s or hemodialys#s or haemodialys#s or hemo-dialys#s or haemo-dialys#s or hemofiltration or haemofiltration or hemo-filtration or haemo-filtration or hemodiafiltration or haemodiafiltration or hemo-diafiltration or haemo-diafiltration).tw. (278398)

7 (CAPD or CCPD).tw. (13729)

8 ((kidney* or renal) adj1 replacement therap*).tw. (17650)

9 or/1-8 (669133)

10 Pulse/ (57856)

11 Pulse Wave Analysis/ (7505)

12 ((pulse or pulsation) adj2 (curve* or tracing* or wave*)).tw. (22791)

13 (PWV or aPWV or BaPWV or cfPWV).tw. (8939)

14 (pulse adj2 (analys$s or velocit* or transit time*)).tw. (13966)

15 Vascular Stiffness/ (10313)

16 ((vascular or aortic or arter*) adj2 (stiffness or stiffening or rigidity)).tw. (17415)

17 ((decreased or reduced or diminished or lessened or lowered) adj3 ((vascular or aortic or arter*) adj compliance)).tw. (951)

18 Blood Flow Velocity/ (85004)

19 ((blood or circulation) adj2 (flow or rate) adj velocit*).tw. (15905)

20 (central adj (pulse or aortic or arterial) adj pressure).tw. (1485)

21 (central pressure or pulse pressure or pulse tension).tw. (15833)

22 AASI.tw. (320)

23 applanation tonomet*.tw. (6198)

24 (SphygmoCor* or Vicorder*).tw. (1354)

25 ((assess* or measur* or determin* or evaluat*) adj3 ((vascular or aortic or arter*) adj elasticit*)).tw. (421)

26 or/10-25 (192443)

27 9 and 26 (7648)

28 exp Animals/ not (exp Animals/ and Humans/) (8740001)

29 27 not 28 (7339)

30 (comment or editorial or interview or letter or news).pt. (2821196)

31 29 not 30 (7163)

32 31 use prmz (2658)

33 chronic kidney failure/ (133258)

34 chronic kidney disease/ (38970)

35 ((kidney* or renal) adj1 (disease* or failure* or impair* or insufficien*)).tw. (397987)

36 (ESRD or ESKD or ESRF or CKD).tw. (55275)

37 exp renal replacement therapy/ (308934)

38 (dialys#s or hemodialys#s or haemodialys#s or hemo-dialys#s or haemo-dialys#s or hemofiltration or haemofiltration or hemo-filtration or haemo-filtration or hemodiafiltration or haemodiafiltration or hemo-diafiltration or haemo-diafiltration).tw. (278398)

39 (CAPD or CCPD).tw. (13729)

40 ((kidney* or renal) adj1 replacement therap*).tw. (17650)

41 or/33-40 (721858)

42 pulse wave/ (13721)

43 ((pulse or pulsation) adj2 (curve* or tracing* or wave*)).tw. (22791)

44 (PWV or aPWV or BaPWV or cfPWV).tw. (8939)

45 (pulse adj2 (analys$s or velocit* or transit time*)).tw. (13966)

46 arterial stiffness/ (10324)

47 ((vascular or aortic or arter*) adj2 (stiffness or stiffening or rigidity)).tw. (17415)

48 ((decreased or reduced or diminished or lessened or lowered) adj3 ((vascular or aortic or arter*) adj compliance)).tw. (951)

49 blood flow velocity/ (85004)

50 ((blood or circulation) adj2 (flow or rate) adj velocit*).tw. (15905)

51 pulse pressure/ (246498)

52 (central pressure or pulse pressure or pulse tension).tw. (15833)

53 AASI.tw. (320)

54 applanation tonomet*.tw. (6198)

55 (SphygmoCor* or Vicorder*).tw. (1354)

56 ((assess* or measur* or determin* or evaluat*) adj3 ((vascular or aortic or arter*) adj elasticit*)).tw. (421)

57 or/42-56 (363761)

58 41 and 57 (16296)

59 exp animal experimentation/ or exp models animal/ or exp animal experiment/ or nonhuman/ or exp vertebrate/ (37664944)

60 exp humans/ or exp human experimentation/ or exp human experiment/ (28628906)

61 59 not 60 (9037681)

62 58 not 61 (14440)

63 (editorial or letter).pt. (2508169)

64 62 not 63 (13980)

65 64 use emczd (3778)

66 32 or 65 (6436)

67 limit 66 to yr=2000-current (5224)

68 remove duplicates from 67 (3751)

69 66 not 67 (1212)

70 remove duplicates from 69 (895)

71 68 or 70 (4646) [total unique refs]

72 71 use prmz (2605) [unique MEDLINE refs]

73 71 use emczd (2041) [unique Embase refs]

***************************
